# Comparative Analysis of Intracellular and *in vitro* Antioxidant Activities of Essential Oil From White and Black Pepper (*Piper nigrum* L.)

**DOI:** 10.3389/fphar.2021.680754

**Published:** 2021-06-25

**Authors:** Ying Wang, Liang Wang, Jin Tan, Rong Li, Zi-Tao Jiang, Shu-Hua Tang

**Affiliations:** Tianjin Key Laboratory of Food Biotechnology, College of Biotechnology and Food Science, Tianjin University of Commerce, Tianjin, China

**Keywords:** *Piper nigrum* L., white pepper essential oil, black pepper essential oil, intracellular antioxidant activities, *in vitro* antioxidant activities

## Abstract

**Ethnopharmacological Relevance:** Pepper essential oils have potential immunomodulatory, anti-tumor, and anti-cancer activities. Pepper exhibits the potential to prevent or attenuate carcinogenesis as therapeutic tools. However, the related mechanism remains unelucidated.

**Aim of the Study:** The present study aims to provide reasonable information for the explanation of the dissimilarity of the essential oils from white (WPEO) and black pepper (BPEO).

**Materials and Methods:** WPEO, BPEO, and their single active component, as well as synthetic antioxidants, were compared by the cell model methods and chemical methods, including intracellular antioxidant activity (CAA), total antioxidant activities (TAA), superoxide radical (SR), hydroxyl radical (HR), DPPH radical (DR) scavenging activities and inhibition ability of lipoprotein lipid peroxidation (ILLP).

**Results:** The median effective concentration (EC_50_) values (mg/mL) of the WPEO and BPEO of SR, HR, DR, and ILLP were 0.437 and 0.327, 0.486 and 0.204, 7.332 and 6.348, 0.688, and 0.624 mg/mL, respectively. The CAA units of WPEO and BPEO were 50.644 and 54.806, respectively. CAA, DR, and TAA of BPEO were significantly higher than those of WPEO (*p* < 0.05). The BPEO and WPEO can be differentiated as the former have higher correlations with 3-carene, α-pinene, β-pinene, and limonene while the latter has a higher caryophyllene correlation. The WPEO and BPEO show a good intracellular scavenging ability of reactive oxygen species in HeLa cells.

**Conclusion:** Generally, pepper oil has stronger activities than single components, indicating that pepper is a broad-spectrum natural antioxidant.

## Introduction

Natural antioxidants in plants are attracting more and more interest due to the global megatrend toward the application of natural additives to food or cosmetics (Alirezalu et al., 2019; Pateiro et al., 2021). Generally, synthetic antioxidants such as butylated hydroxytoluene (BHT), butylated hydroxyanisole (BHA), and propylgallate (PG) have been applied to free radical deactivation and lipid oxidation control at a lower cost than natural alternatives (Xu et al., 2021). However, these synthetic antioxidants pose potential risks to human health (Alizadeh et al., 2019). Consequently, many studies have focused on herbs and spices which are rich in antioxidant compounds. Spices are usually regarded as non-perishable products, and thus some of them could be applied to food preservation due to their antimicrobial and antioxidant properties (Garcia et al., 2018).

Black pepper is one of the most popular worldwide spices and is known as “The King of Spices” or “Black Gold” because of its pungency, aromatic odor, and flavor (Wei et al., 2019). It is dried from the unripe and green berries of *Piper nigrum* L. with its outside pericarp, while white pepper is the white inside produced from fully ripe berries with the removing of the pericarp (Chithra et al., 2011). White pepper is preferred to black pepper in some countries and regions due to its white color for the light-colored food preparations (Chithra et al., 2011). For example, in Indonesia, half of the peppers convert to white, and people think white pepper is the value-added form of black pepper with decortication/pericarp removal, lighter color, and thus higher quality (Vinod et al., 2014). Despite these beliefs the question remains, is white pepper really better than black pepper?

There are both intrinsic and extrinsic differences between the two peppers. Besides their colors, harvesting time, and processing modes (Prabhu et al., 2015), peppers contain plentiful bioactive compounds including lignans, alkaloids (piperine), flavonoids, polyphenols, amides, and aromatic compounds (Jin et al., 2013; Rathod and Rathod, 2014; Wang et al., 2018). Many studies have focused on the differences between white and black peppers. Garcia et al. (2018) revealed the antifungal capacity differences between white and black peppers with 76.3 and 77% regarding average fungal infection, respectively. Agbor et al. (2006) reported that black pepper contained a significantly higher content of polyphenols and exhibited more effective free radical and reactive oxygen species scavenging activities than white pepper. Olalere et al. (2018) found that the oleoresin extracts from white pepper had higher inhibition abilities toward the DPPH and ABTS free radicals than black pepper extract. Piperine and phenolics contribute to the pungency of peppers (Srinivasan, 2007), while the characteristic aroma and flavor of peppers are attributed to essential oils (Wei et al., 2019). As the highly important substances of aroma and flavor for spices, essential oils are always featured by high antimicrobial and antioxidant activities (Teixeira et al., 2013). However, though the current scientific research compares the differences between the two peppers from the above points of view, only limited data has revealed the essential oils of white and black peppers and their antioxidant activities. (Abd El Mageed et al., 2011) studied the effect of microwaves on the essential oils of white and black pepper and their antioxidant activities from the views of the inhibiting effect for the oxidation of linoleic acid and DPPH radical scavenging. However, they did not explain why black pepper was different from white pepper. Li et al. (2020) recently differentiated black and white pepper in five provinces of southern China. The chemical components and biological activities of essential oils from these peppers were investigated and they found that black pepper is slightly better than white pepper in terms of antioxidant activity. Singh et al. (2013) reported the antioxidant and antimicrobial potentials of essential oil and the oleoresins of white pepper without comparing it to black pepper. Thus, systematic, comprehensive, and comparative studies of the intracellular and *in vitro* antioxidant activities and free radical scavenging potential of the essential oils of the two peppers and the rational explanation are worthy of studying. In addition, essential oil is a mixture of multiple bio-active compounds, and whether synergistic effects enhance bio-activities over any single compound should be further studied. Therefore, the present study aims to 1) compare the intracellular and *in vitro* antioxidant activities of essential oils from white (WPEO) and black pepper (BPEO), including intracellular antioxidant activity (CAA), total antioxidant activities (TAA), superoxide radical (SR), hydroxyl radical (HR), DPPH radical (DR) scavenging activities and the inhibition ability of lipoprotein lipid peroxidation (ILLP), 2) compare them with their single active component and the synthetic antioxidants, butylated hydroxytoluene (BHT), propylgallate (PG) and ascorbic acid (Vc), and 3) relate the differences between the two peppers with their main components and explain whether white pepper is better than black pepper and why. Although (Li et al., 2020) recently studied the chemical components and biological activities of essential oils from five provinces of southern China, they focused more on the influence of the geographical origins of pepper and how this relates to their chemical composition and bioactivities. Unlike other literature on this subject, we undertake a comprehensive comparison between the two peppers and offer reasonable information for the explanation of the dissimilarity, which is important for a better understanding of pepper as a broad-spectrum natural spice and antioxidant with important prospects and applications.

## Materials and Methods

### Plant Materials and Essential Oil Extraction

The berry fruits of white and black peppers were collected from Hainan province in China and identified by Professor Junbo Xie from Tianjin University of Traditional Chinese Medicine, China. The voucher specimens have been deposited in the Food Analysis Laboratory, College of Biotechnology and Food Science, Tianjin University of Commerce (voucher no. 201801 and 201802). The reference specimens are stored in the Herbarium, Institute of Botany, CAS (Ref. Barcode: 00125120 and 00125121). The fruits were ground with a blade homogenizer (IKA-A10, Germany), sieved to 40 mesh, and stored in a cool and dry place. WPEO and BPEO were then extracted by green and solvent-free simultaneous ultrasonic-microwave-assisted hydrodistillation extraction using the ultrasonic and microwave extracting apparatus (CW-2000, Shanghai Xintuo Microwave Instrument Co. Ltd., China). The extraction conditions followed Wang et al. (2018), and the extraction yields of WPEO and BPEO were 4.1 ± 0.1 and 4.0 ± 0.1%, respectively.

### Chemicals

The essential oil standards (α-pinene, β-pinene, 2-carene, 3-carene, limonene, linalool, and caryophyllene), BHT, PG, Vc, pyrogallol, thiobarbituric acid (TBA), trichloroacetic acid (TCA), dimethyl sulfoxide (DMSO), and 2,2-diphenyl-1-picrylhydrazyl (DPPH), etc. were purchased from Sigma and Sigma-Aldrich Company, United States. Other chemicals used were of analytical reagent grade. The fetal bovine serum (FBS) and Dulbecco’s modified eagle’s medium (DMEM) were obtained from Gibco Co., United States. Double-antibody (penicillin-streptomycin) was purchased from HyClone Co., United States. Methyl thiazolyl tetrazolium (MTT) was purchased from Solarbio Biotechnology Co., Beijing, China. 2′,7′-Dichlorofluorescein diacetate (DCFH-DA) assay kit was bought from Beyotime Institute of Biotechnology, Shanghai, China. Distilled water was used throughout.

### Methods

The antioxidant activity and free radical scavenging potential of one studied sample cannot be evaluated comprehensively by a single testing method (Wang et al., 2014), and thus a combination of cell models and different chemical methods with a variety of *in vitro* assays was applied to compare the intracellular and *in vitro* antioxidant activities of WPEO and BPEO. The antioxidant activities and free radical scavenging potential of the essential oils were also compared with the standards of their components (Wang et al., 2018) and well-known synthetic antioxidants such as BHT, PG, and Vc.

#### Intracellular Antioxidant Activity

HeLa cells were obtained from the Institute of Biochemistry and Cell Biology (Shanghai, China), and the *mycoplasma* testing method (Young et al., 2010) was used to ensure that no *mycoplasma* contamination occurs. Methods by both Schantz et al. (2010) and Yang et al. (2013)were used with some modifications to measure the cell viability and intracellular antioxidation. Briefly, HeLa cells were transferred into a 96-well plate with 100 μL per well of DMEM medium at a density of 1 × 10^5^/mL. The Hela cells were then incubated with 5% CO_2_ for 24 h at 37°C. The medium was discarded and the cells were carefully washed by autoclaved PBS (pH = 7.4) thrice and followed by the treatment with 100 μL different concentrations of the essential oils (WPEO or BPEO) or their standards (α-pinene, β-pinene, 2-carene, 3-carene, limonene, linalool or caryophyllene) for another 24 h. Different concentrations (0.001–2 μL/mL) of essential oils and standards were dissolved in different concentrations of DMSO, i.e., 0.001, 0.01 and 0.1 in 0% DMSO, 0.2 in 2% DMSO, 0.4 in 4% DMSO, 0.5 in 5% DMSO, 1 in 10% DMSO, 2 in 20% DMSO, respectively. Therefore, the corresponding concentrations of DMSO were used as solvent controls for the cell viability and intracellular antioxidation experiments. The growth medium was used to replace the oils and the standards in the control well. After repeated washing, 5 mg/mL of MTT was added for another 4-h incubation, and 150 μL of DMSO was then added to dissolve the purple crystals. The absorbance at 570 nm was then measured.

In the intracellular antioxidation assay, the cells were transferred and incubated according to the same procedures in the above cell viability assay. An aliquot of 100 μL of 10 μmol/L DCFH-DA was mixed with 100 μL of 0.1 μL/mL oils or the standard solution for 1 h. After the wells were carefully washed by PBS (pH = 7.4), each well was added with 100 μL of 200 μmol/L H_2_O_2_. The fluorescence intensities were measured at every 5 min during the 1-h incubation period with the excitation and emission wavelengths at 525 and 488 nm, respectively. The integral area of the relative fluorescence intensity of the sample was ∫SA, while the integral area of the relative fluorescence intensity of the control with the only medium was ∫CA. The blank was prepared without both the oils and H_2_O_2_ solution. The intracellular antioxidant activity CAA unit was calculated according to the following equation: CAA (%) = (1–∫SA/∫CA) × 100% (Wolfe and Liu, 2007).

#### Total Antioxidant Activities

The phosphomolybdenum complex method was applied to evaluate the total antioxidant activities (Ozkan et al., 2007). Briefly, 0.4 mL WPEO, BPEO, standards or synthetic antioxidants (BHT and PG) solution of different concentrations dissolved in 100% ethanol or Vc dissolved in distilled water was added into 4 mL phosphorus molybdenum reagents (28.0 mmol/L sodium phosphate, 0.6 mmol/L sulfuric acid, and 4.0 mmol/L ammonium molybdate). The reactions were carried out at 95°C for 1.5 h. The absorbance at 695 nm was measured after cooling down.

#### Superoxide Radical, Hydroxyl Radical, and DPPH Radical Scavenging Activities

Superoxide radical (SR), hydroxyl radical (HR), and DPPH radical (DR) scavenging activities of WPEO, BPEO, standards, or synthetic antioxidants (BHT, PG, or Vc) were measured according to our previous work (Wang et al., 2014) and based on the pyrogallol autoxidation method (Gulcin et al., 2010), the benzoic acid fluorescence method (Chen et al., 2002) and the method described by Zhan et al. (2006) with slight modifications, respectively.

#### Inhibition of Lipoprotein Lipid Peroxidation

The inhibition of lipoprotein lipid peroxidation activity (ILLP) was measured by the method described by Pandey et al. (2007) with slight modifications using homogenates of egg yolk as the lipid-rich media. Egg yolk homogenate (0.4 mL, dissolve in 0.1 mol/L PBS, pH = 7.5, 2%, v/v) was added with 0.1 mL WPEO, BPEO, standards or synthetic antioxidants (BHT, PG or Vc) solution of different concentrations, 0.4 mL FeSO_4_ (25.0 mmol/L, dissolved in 1% H_2_ SO_4_), and then the final volume was made up to 4 mL by PBS. The above mixture was shaken in a water bath at 37 °C for 30 min to induce lipid peroxidation. 1 mL of 20% TCA (w/v) was then added, well mixed, and reacted for 10 min. After centrifugation (4,000 r/min, 12 min), 4 mL of the supernatant was mixed with 2 mL 0.28% TBA (w/v) and reacted and boiled for 15 min. After cooling, the absorbance at 532 nm was measured. The control was a substitution of the sample with 0.1 mL of ethanol or distilled water. ILLP was calculated by the following equation: ILLP (%) = (Ac–As)/Ac × 100% (Ac is the absorbance of the control, while As is the absorbance of the sample).

#### Statistical Analyses

Means and standard deviations (SD) of the triplicates for all samples were calculated. All statistical analyses were done by SPSS (Statistical Package for Social Science) Version 16.0. The antioxidant activities and free radical scavenging potential of WPEO, BPEO, their standards, and synthetic antioxidants at the same concentration were compared by one-way ANOVA. Probit analysis was applied to calculate the median effective concentration (EC_50_) values of the essential oils and the synthetic antioxidants. The higher the EC_50_ value, the lower the antioxidant and free radical scavenging activities (Gulcin et al., 2010). The ratios of seven standards to essential oils in terms of different antioxidant and scavenging activities were subject to principal component analysis (PCA), which was calculated using PRIMER 6 software package version 6.1.5 (Plymouth Marine Laboratory, United Kingdom). The PCA analysis was also carried based on DPPH radical scavenging activities and superoxide radical scavenging activities of WPEO and BPEO in the present study and five different habitats reported by Li et al. (2020).

## Results and Discussion

The chemical compositions of WPEO and BPEO were extracted by ultrasonic-microwave assisted extraction (UMAE). They were then analyzed by GC-MS in our previous study (Wang et al., 2018). The information including the structures and relative contents of the seven standards (α-pinene, β-pinene, 2-carene, 3-carene, limonene, linalool, and caryophyllene) used in the present study are shown in Supplementary Table S1. Caryophyllene and 3-carene are the most abundant component of WPEO and BPEO, respectively.

### Intracellular and *in vitro* Antioxidant Activities and Free Radical Scavenging Potential of White Pepper Essential Oil and Black Pepper Essential Oil

#### Cell Viability

**FIGURE 1:**
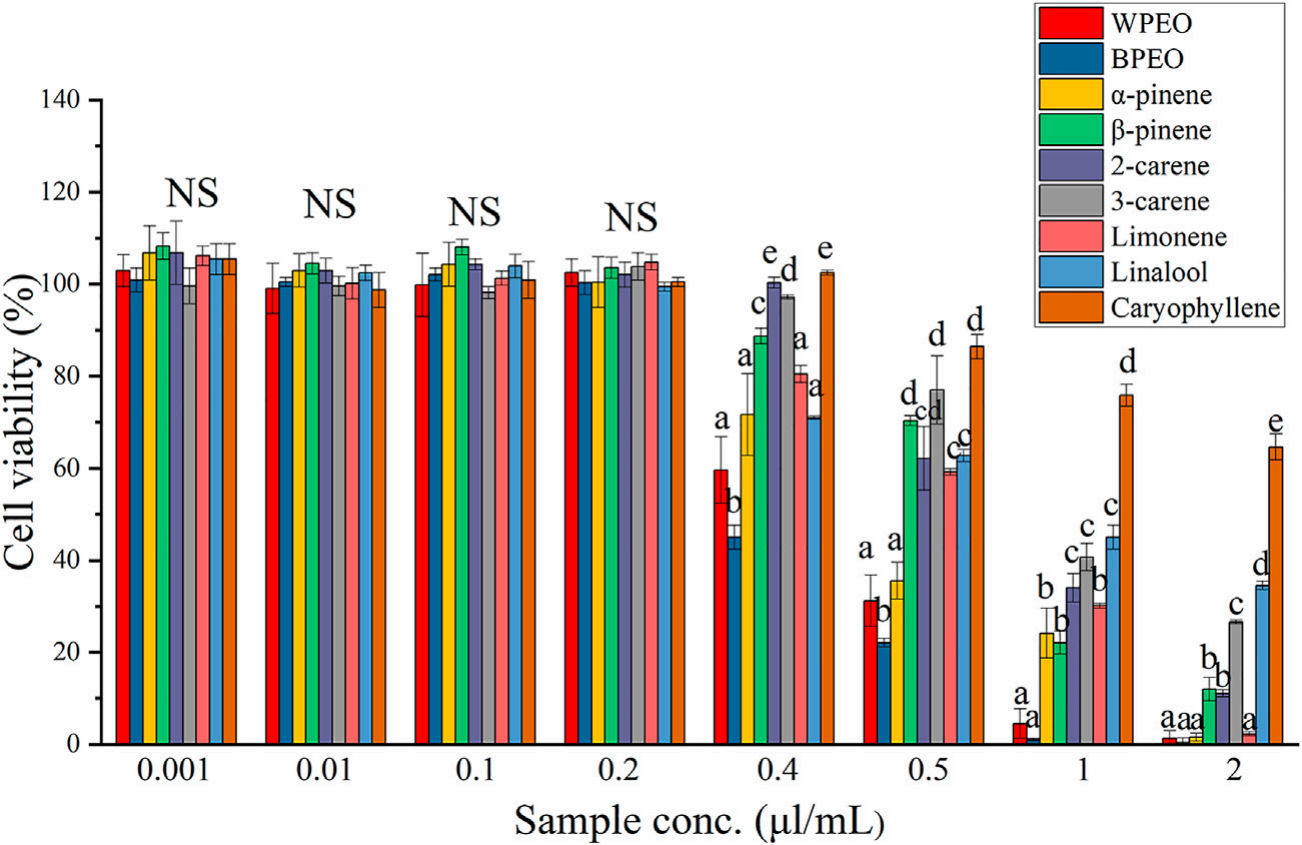
Effects of WPEO, BPEO, and seven standards on cell viability in HeLa cells. (Data are reported as the mean ± SD of three replicates. Bars with different letters are significantly different at *p* ≤ 0.05 according to one-way ANOVA; NS, not significant difference; WPEO, white pepper essential oil; BPEO, black pepper essential oil).

MTT, a yellow tetrazolium salt, could be reduced by succinodehydrogenase in the living cells to a purple formazan, which can be dissolved by DMSO and absorbance at 570 nm reflects indirectly the number of the living cells (Adach et al., 2016; Sakulnarmrat et al., 2013). The effects of WPEO, BPEO, and seven standards with different concentrations on cell viability in HeLa cells are shown in Figure 1. No significant difference was observed among WPEO, BPEO, and standards with the concentration ranged from 0.001 to 0.2 μL/mL. They did not cause a significant effect on the cell viability at concentrations lower than 0.2 μL/mL, while the cell viability was reduced when the concentration increased to 0.4 μL/mL. The obvious inhibition of the essential oils and the standards on HeLa cells then occurred when the concentration further increased from 0.4 to 2 μL/mL. The inhibitory effect of WPEO seems slightly lower than that of BPEO on HeLa cells, but both WPEO and BPEO inhibited the cell viability more severely than all the standards. Caryophyllene exhibited the lowest inhibition effects on HeLa cells. The growth of HeLa cells is inhibited by WPEO or BPEO, indicating the pepper essential oils can play an anticancer role in inhibiting HeLa cells when their concentrations are up to 0.4 μL/mL. Previous research has also shown that black pepper extracts have potential immunomodulatory, anti-tumor, and anti-cancer activities, indicating pepper that pepper exhibits the potential to prevent or attenuate carcinogenesis as therapeutic tools (Majdalawieh and Carr, 2010). The essential oils from *piper klotzschianum*, *P. hispidum*, and *P. arboreum* all showed the best cytotoxic activity, inhibiting murine melanoma cell lines and human hepatocellular carcinoma and promyelocytic leukemia (Lima et al., 2019). The essential oils of other plants such as *Juniperus oxycedrus* L. presented anti-tumor properties in breast cancer cells due to the high level of α-pinene, β-pinene, limonene, and 3-carene (El-Abid et al., 2019). α- and β-pinenes are inhibitors in breast cancer, and *in vitro* present cytotoxic activity against human cancer cells, instead of healthy cells including red blood cells or even whole organisms (Mercier et al., 2008). Although exhibiting the lowest inhibition effects on HeLa cells in the present study, caryophyllene still showed anti-proliferative properties in cancer cells (Mboge et al., 2019). However, although Lampronti et al. (2006) did not find an anti-tumor effect of the individual α or β-pinenes, they believed that possible synergistic effects with other monoterpenes and sesquiterpenes such as caryophyllene could exist. All these studies provide further insight into the potential applications of terpenes and a mixture of terpenes, i.e., the essential oils as potential anti-tumor agents. Besides the anticancer role of WPEO and BPEO in killing HeLa cells, we focused on the intracellular antioxidant activities.

#### Intracellular Antioxidant Activity

Although the traditional chemical methods to evaluate antioxidant activities *in vitro* are quick, simple, cheap, and convenient, they neither truly simulate the internal physiological environment of the human body, nor accurately reflect the antioxidant capacity of the antioxidants *in vivo*. Besides, the animal models and human experiments are expensive and time-consuming, while the cell model method provides an economical and rapid method for better antioxidant research. Compared with the chemical methods, the cell model method has a better biological correlation and might predict the antioxidant activity of the antioxidants in the human body more accurately. Therefore, within the non-cytotoxic range lower than 0.2 μL/mL on HeLa cells, HeLa cells were chosen to simulate an *in vivo* environment to investigate the intracellular antioxidant activities of the essential oils and their standards with concentration at 0.1 μL/mL. Non-fluorescent DCFH-DA could enter cells freely and then be hydrolyzed by intracellular esterases to form DCFH (Labieniec and Gabryelak, 2007). Abundant cellular ROS could be simulated by H_2_O_2_, and then the produced ROS could oxidize the non-fluorescent DCFH to fluorescent DCF, therefore, the measurement of fluorescence intensity reflects the cellular ROS directly (Qian et al., 2012; Xu and Chang, 2012). When antioxidants possess the scavenging ability of ROS, the oxidation of DCFH could be inhibited or even eliminated, and thus the fluorescence intensity of DCF would decrease.

**FIGURE 2:**
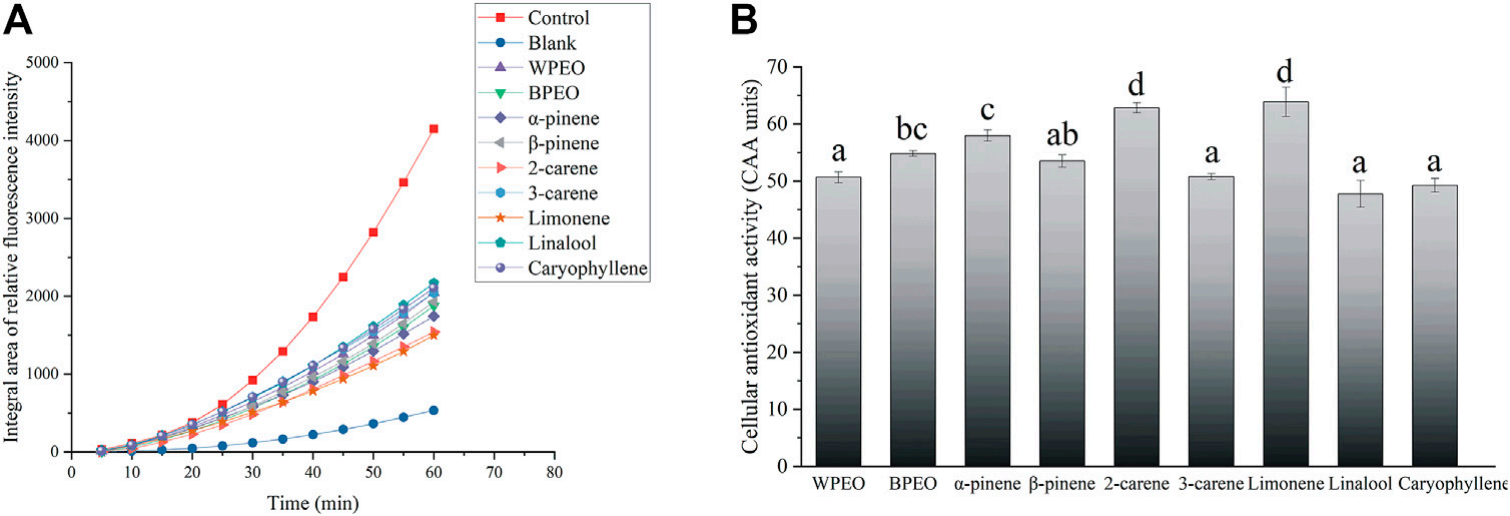
The integral areas of relative fluorescence intensity **(A)** and the intracellular antioxidant activity (CAA) of WPEO, BPEO, and seven standards **(B)** in HeLa cells. (Data are reported as the mean ± SD of three replicates. Bars with different letters are significantly different at *p* ≤ 0.05 according to one-way ANOVA; WPEO, white pepper essential oil; BPEO, black pepper essential oil).

The integral areas of the relative fluorescence intensity of WPEO, BPEO, and seven standards in HeLa cells are shown in Figure 2A. After 1-h cultivation, the relative fluorescence intensity in the control group stimulated by H_2_O_2_ was nearly eight times that of the blank group without the H_2_O_2_ stimulation. The relative fluorescence intensities of WPEO, BPEO, and seven standards were all significantly lower than the control, indicating that all of them had the intracellular ROS scavenging activity in HeLa cells. The intracellular antioxidant activity (CAA) of WPEO, BPEO, and seven standards are also shown in Figure 2B. BPEO showed significantly higher CAA than WPEO, while for the standards the order was limonene = 2-carene > α-pinene > β-pinene > 3-carene = caryophyllene = linalool. Limonene, α-pinene, β-pinene, and 3-carene all have higher contents in BPEO than WPEO, and they account for about 75% in BPEO and play a role in scavenging intracellular ROS, leading to the higher CAA of BPEO (Supplementary Table S1). Although 2-carene shows high CAA but lower content in BPEO than WPEO, it only accounts for 1.1 and 0.1% in WPEO and BPEO, respectively, (Supplementary Table S1). The essential oils rich in monoterpenes, oxygenated monoterpenes, and/or sesquiterpenes generally show greater and higher antioxidative potential (Tepe et al., 2004; Deba et al., 2008). In particular, the monoterpenes found in these essential oils may act as radical scavenging agents, as the monoterpenes are very reactive because of the presence of C=C, a double bond between two atoms of carbon (Mercier et al., 2009). In addition, cyclic monoterpenes will preferentially separate from an aqueous phase and thus easily access cell membrane structures due to their lipophilic character (Sikkema et al., 1994). On the other hand, as sesquiterpene hydrocarbons, caryophyllene subjected to antioxidant study always shows a low antioxidant effect (Ruberto and Baratta, 2000).

The activities of synthetic antioxidants have only been examined by chemical methods, while they have not been examined by cell model. Compared with animal models and human experiments, the cell model method is cheap and rapid. However, compared with the chemical methods, the cell model method is relatively expensive and time-consuming. The results from chemical methods, to some extent, could reflect the comparisons between peppers and synthetic antioxidants. In addition, the activities of the pepper essential oils and their components are what we are more interested in. Therefore, the activities of synthetic antioxidants have only been evaluated with chemical methods and without cell model methods.

#### Total Antioxidant Activities

**FIGURE 3:**
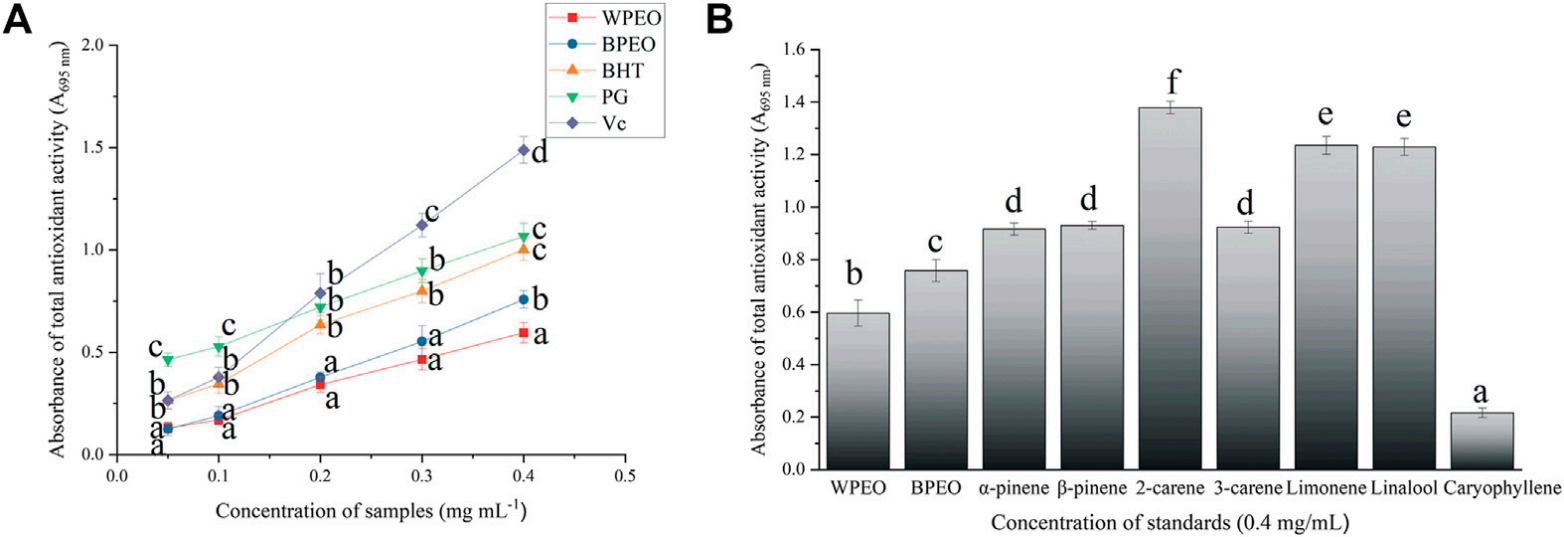
The total antioxidant activities (TAA) of WPEO, BPEO, and synthetic antioxidants (BHT, PG, and Vc) **(A)** and seven standards **(B)**. (Data are reported as the mean ± SD of three replicates. Bars and lines with different letters are significantly different at *p* ≤ 0.05 according to one-way ANOVA; WPEO, white pepper essential oil; BPEO, black pepper essential oil; BHT, butylated hydroxytoluene; PG, propylgallate; Vc, ascorbic acid).

The capacities in reducing Mo^6+^ to Mo^5+^ of the antioxidants stand for TAA, which reaction produces a blue-green phosphomolybdenum complex with the maximum absorption wavelength at 695 nm (Merugu et al., 2021). The TAA results of WPEO, BPEO, and synthetic antioxidants (BHT, PG, and Vc) are shown in Figure 3A. In all antioxidants, TAA followed the same trend, i.e., increased rapidly with the sample concentrations; however, the increasing rates varied among different antioxidants. Although the TAA of both pepper essential oils was lower than those of the three synthesized antioxidants, WPEO and BPEO still showed significant and concentration-dependent TAA. The TAA of WPEO, BPEO, and seven standards are also shown in Figure 3B. BPEO showed a significantly higher TAA than WPEO, and the TAA declined in the trend of 2-carene > limonene = linalool > α-pinene = β-pinene = 3-carene > caryophyllene. Limonene, α-pinene, β-pinene, and 3-carene with relatively higher TAA exhibiting higher contents in BPEO than WPEO, while caryophyllene, with the lowest TAA, is significantly richer in WPEO than BPEO, which leads to the higher TAA of BPEO than WPEO (Figure 3). This trend of TAA among essential oil and standards is similar to the trends of CAA, which is BPEO > WPEO and caryophyllene has the lowest values (Figure 2). As a sesquiterpene, caryophyllene always has the lowest CAA or TAA likely due to its structure with higher molecule weight and more steric hindrance than those small molecule monoterpenes, which might work more effectively as reactive and intercellular ROS scavenging antioxidants (Mukai et al., 2018).

#### Superoxide Radical Scavenging Activity

Among all the ROS, superoxide anion radical is one of the strongest free radicals. SR results, measured by the autoxidation of the pyrogallol method of WPEO, BPEO, and synthetic antioxidants (BHT, PG, and Vc) are shown in Supplementary Figure S1A. For all antioxidants, SR showed the same trend, i.e., it increased rapidly with the sample concentrations. However, the rates of increase differed among the antioxidants. WPEO and BPEO showed significant and concentration-dependent SR, which are similar, a little higher and 2-fold as that of Vc, BHT, and PG, respectively. The SR of WPEO, BPEO, and seven standards are also displayed in Supplementary Figure S1B. The EC_50_ values (mg/mL) of all samples and standards are shown in Table 1. High EC_50_ values indicate low scavenging activities, and thus the order of SR was BPEO > Vc > WPEO > BHT > PG, and SR among the oils and standards declined in the trend of limonene > BPEO > 3-carene > WPEO > 2-carene > β-pinene > α-pinene > linalool > caryophyllene (Table 1).

**TABLE 1:** EC_50_ values (mg/mL) of WPEO, BPEO, synthetic antioxidants (BHT, PG, and Vc) and seven standards (SR, superoxide radical scavenging activity; HR, hydroxyl radical scavenging activity; DR, DPPH radical scavenging activity; ILLP, inhibition of lipoprotein lipid peroxidation; WPEO, white pepper essential oil; BPEO, black pepper essential oil; BHT, butylated hydroxytoluene; PG, propylgallate; Vc, ascorbic acid).

Antioxidants		EC_50_ values (mg/mL)			
		SR	HR (× 10^-5^)	DR	ILLP
Natural	WPEO	0.437	0.486	7.332	0.688
	BPEO	0.327	0.204	6.348	0.624
Synthetic	BHT	0.591	0.371	2.594	0.018
	PG	1.256	1.564	0.719	0.125
	Vc	0.401	7.297	1.416	1.121
Standards	α-pinene	0.550	0.843	10.201	0.819
	β-pinene	0.473	1.169	10.272	1.025
	2-carene	0.457	2.173	10.274	1.094
	3-carene	0.412	0.710	10.062	0.751
	Limonene	0.291	4.490	9.720	0.848
	Linalool	0.637	0.557	10.414	0.619
	Caryophyllene	0.727	0.779	10.554	0.786

#### Hydroxyl Radical Scavenging Activity

Hydroxyl radical scavengers play a significant role in biological systems due to the high activity of hydroxyl radicals. All samples studied here exhibited HR in a dose-dependent manner to varying degrees (Supplementary Figure S2A). BPEO and WPEO presented stronger HR than the synthetic antioxidants, BHT, and even several-folds as Vc. HR of WPEO, BPEO, and seven standards are also illustrated in Supplementary Figure S2B. The EC_50_ results showed that HR declined in the trend of BPEO > BHT > WPEO > PG > Vc, and the HR order among the oils and standards was BPEO > WPEO > linalool > 3-carene > caryophyllene > α-pinene > β-pinene > 2-carene > limonene (Table 1). Compared with the synthetic antioxidants and single standard, both WPEO and BPEO are the most effective in removing OH.

#### DPPH Radical Scavenging Activity

Antioxidants always result in a decrease in absorbance at 517 nm by the reaction of DPPH radicals scavenging. DPPH assays are simply chemical assays and may only be used to define the chemical profile of a preparation. However, the application of this quick and easy chemical assay combined with other pharmacological experiments, to some extent, could help reflect the differences between peppers and synthetic antioxidants. Supplementary Figure S3A exhibits the DR results of WPEO, BPEO, and synthetic antioxidants (BHT, PG, and Vc). The scavenging activity increased with the concentration from 1 to 5 mg/mL of WPEO and BPEO, though they showed lower DR than the synthetic antioxidants. The EC_50_ results also showed PG > Vc > BHT > BPEO > WPEO (Table 1). On the other hand, the EC_50_ results showing the DR order of oils and standards followed the decreasing trend of BPEO > WPEO > limonene > 3-carene > α-pinene > β-pinene > 2-carene > linalool > caryophyllene (Table 1). The most abundant component in BPEO, 3-carene, was significantly stronger than caryophyllene, the major component in WPEO, indicating that BPEO was more DR active than WPEO. Furthermore, DR of both WPEO and BPEO were better than those of the single standards in removing DPPH radicals.

#### Inhibition of Lipoprotein Lipid Peroxidation

Lipid peroxidation could lead to cell damage and different oxidant reactions in organisms. Among the two essential oils and the three synthetic antioxidants, the ILLP followed the declining order of BHT > PG > BPEO > WPEO > Vc, and they were all concentration-dependent (Supplementary Figure S4A; Table 1). The EC_50_ results of oil samples and standards showed that ILLP followed the decreasing trend of linalool > BPEO > WPEO > 3-carene > caryophyllene > α-pinene > limonene > β-pinene > 2-carene (Supplementary Figure S4B; Table 1).

All the above orders, although based on different activities, followed a similar trend, with BPEO always having higher activities than WPEO, and sometimes the essential oils had higher capacities than the synthetic antioxidants, which showed good evidence for future research on natural antioxidants. BPEO and WPEO were generally better than those of the single standards in different aspects.

### Principal Component Analysis

**FIGURE 4:**
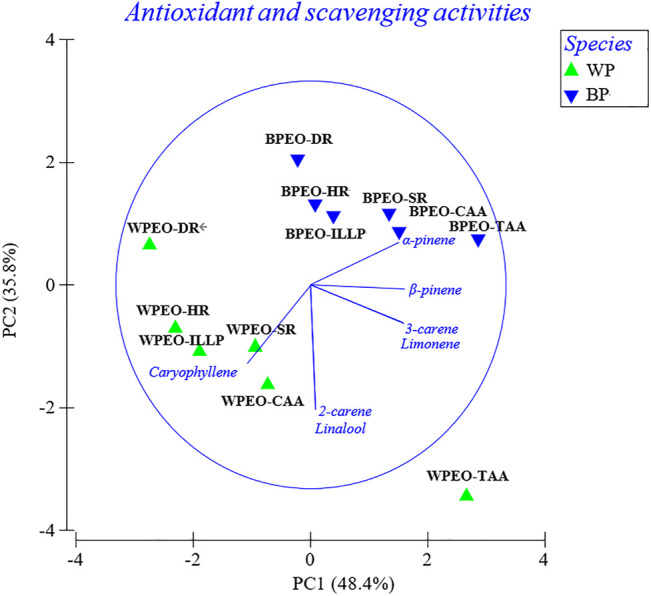
PCA plot showing the grouping of WPEO and BPEO based on the ratio of seven standards to essential oils in terms of different antioxidant and scavenging activities (CAA, intracellular antioxidant activity; TAA, total antioxidant activities; SR, superoxide radical scavenging activity; HR, hydroxyl radical scavenging activity; DR, DPPH radical scavenging activity; ILLP, inhibition of lipoprotein lipid peroxidation; WPEO, white pepper essential oil; BPEO, black pepper essential oil; the symbols in same shapes stand for the same species).

The PCA plot shows the grouping of WPEO and BPEO based on the ratio of seven standards to essential oils in terms of different antioxidant and scavenging activities (Figure 4). WPEO and BPEO could be differentiated from the PCA plot based on the different components and scavenging activities (Figure 4). The differentiation between BPEO and WPEO is due to the fact that the former has higher correlations with 3-carene, α-pinene, β-pinene, and limonene while the latter has a higher caryophyllene correlation.

### White and Black Pepper, Which one is Better?

Essential oils are antioxidants that are often characterized by reducing power and scavenging activities (Pateiro et al., 2021). In the present study, essential oils from the white and black peppers both show significant abilities to scavenge free radicals such as superoxide, hydroxyl, and DPPH radicals, and on some occasions, the antioxidant activities of WPEO and BPEO are even higher than the synthetic antioxidants (BHT, PG, and Vc). The ability to scavenge the ROS of white and black peppers are beneficial to human health (Abd El Mageed et al., 2011), but which one is better? Many previous studies have focused on the differences between white and black peppers from other points of view as we mentioned in the introduction, however, we also care about essential oils that contribute to the aroma and flavor of peppers.

The data in the present study reveal insights about the essential oils of white and black peppers and their antioxidant activities and explains why they are different. Generally, BPEO always exhibited higher activities than WPEO. Similarly, Li et al. (2020) reported that BPEO was overall slightly better than WPEO in the antioxidant activity field by DPPH and ABTS radical scavenging assay, and ferric reducing power assay. The scavenging abilities of 3-carene are more effective than those of caryophyllene, which could explain the reason why the scavenging potentials of BPEO are higher than WPEO in the present study since 3-carene and caryophyllene are the most abundant components of BPEO and WPEO, respectively. It seems to be a general trend that the essential oils that contain monoterpene hydrocarbons and/or oxygenated monoterpenes have a higher antioxidative potential, due to the presence of a double bond between two atoms of carbon leading to very reactive volatile monoterpenes (Tepe et al., 2004; Mercier et al., 2009). 3-carene is a bicyclic monoterpene, which is a popular component of essential oils with strong antioxidant/antifungal activity (Kang et al., 2019; Li et al., 2020). In the present study, caryophyllene as sesquiterpene hydrocarbons always showed low activities, similar to the conclusions of Ruberto and Baratta (2000) and it is necessary to draw the opposite conclusion to other studies. Myszka et al. (2017) reported that WPEO was better than BPEO in suppressing the spoilage activity of *Pseudomonas fluorescens* KM06 in fresh-cut lettuce due to the higher content of caryophyllene.

It is not rational to only consider the activities of a single standard. Pepper essential oil is a complex mixture and always has stronger antioxidant activities and free radical scavenging potentials than their highest single standards. For example, Li et al. (2020) suggested that 3-carene, limonene, and caryophyllene reached about 80% of the total proportion of pepper essential oil, and thus were the main active ingredients. Linalool, a tertiary allylic alcohol, also showed a pro-oxidant effect and contributed to the total antioxidant activities of essential oils (Ruberto and Baratta, 2000). In addition, the effects of α-pinene vary depending on the composition of monoterpenes and sesquiterpenes, and there might be synergistic effects among the compounds on the total biological effect of the essential oil (Sonboli et al., 2006). An explanation for the contradictions in research could be that the Gram-positive bacteria and yeast tested in those studies were sensitive to the essential oil of *Cordia verbenacea* D.C., whose main constituents were α-pinenes, however, Gram-negative species were resistant (de Carvalho Jr et al., 2004). However, other studies have reported the antibacterial effect of these terpenes on both Gram-negative and Gram-positive bacteria as well as a strong antifungal activity (Martins et al., 2003). The observation that these non-phenolic based antioxidants sometimes exhibit non-concentration-dependent effects might be ascribed to the fact that they work cooperatively in the oil complex mixtures with synergistic effects. Since the differences between white and black pepper only lie in the degree of the ripeness of the berries and the presence or absence of the pericarp, we should consider whether generalizing and classifying peppers into the categories of white and black is too simple.

**FIGURE 5:**
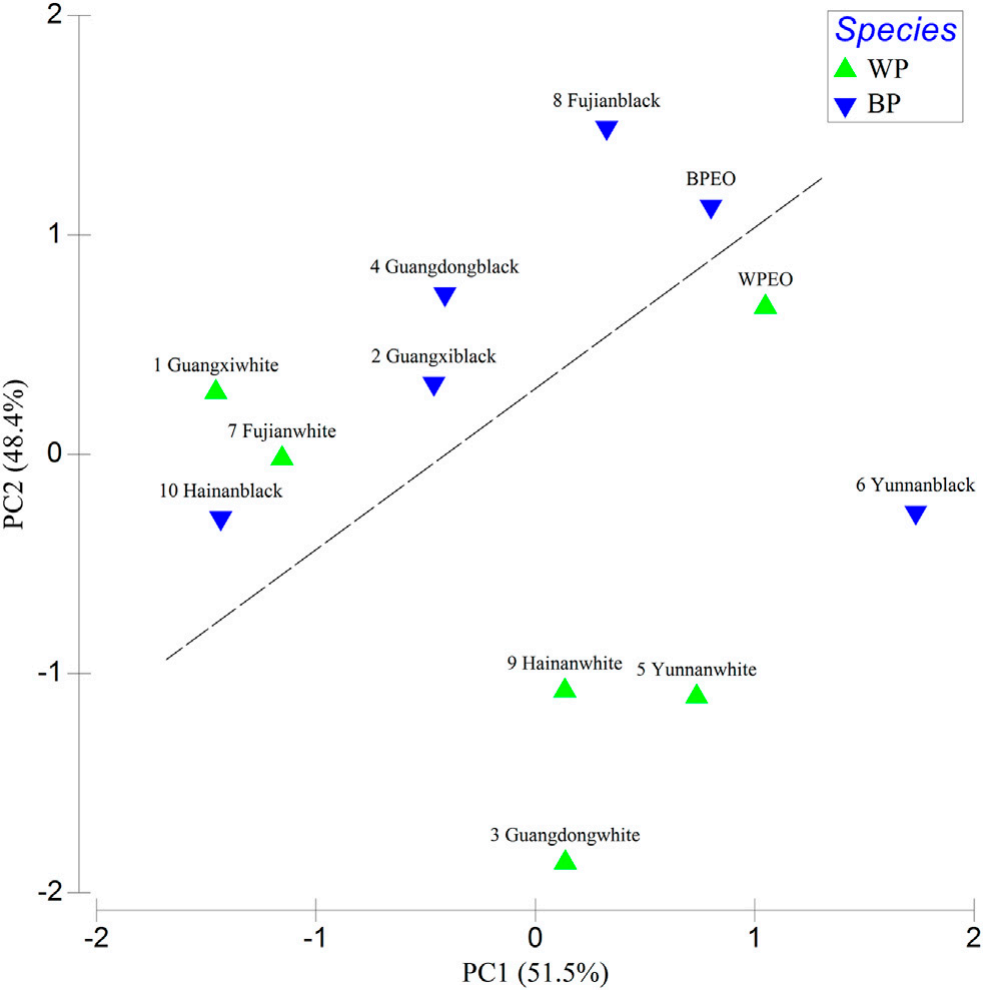
PCA plot based on the DPPH radical scavenging activities (DR) and superoxide radical scavenging activities (SR) of the essential oils from white pepper and black in the present study and five different habitats reported by Li et al. (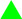: White pepper; 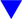: Black pepper; WPEO and BPEO in this study; 1 (Guangxi white), 2 (Guangxi black), 3 (Guangdong white), 4 (Guangdong black), 5 (Yunnan white), 6 (Yunnan black), 7 (Fujian white), 8 (Fujian black), 9 (Hainan white), and 10 (Hainan black) in Li et al.’s study^[18]^; WPEO, white pepper essential oil; BPEO, black pepper essential oil).

It would be better to analyze a series of different pepper samples for future work. The PCA analysis in this study was carried out based on the DPPH radical scavenging activities and superoxide radical scavenging activities of WPEO and BPEO and five different habitats were reported by Li et al. (2020) (Figure 5). The ten white and black pepper samples in Li’s study, together with the WPEO and BPEO samples in our study, were roughly differentiated from the PCA plot based on the DR and SR activities as black and white, except for three outliers (Figure 5). The outliers are two white peppers with high DR activity, namely Guangxiwhite and Fujianwhite, and one black pepper with low DR activity, namely Yunnanblack. Although our berry fruits of white and black peppers were also collected from Hainan province, they seem far away from Hainanwhite and Hainanblack in Li’s study. One of the possible explanations is that, in addition to species, essential oil composition and their antioxidant activities vary depending on the geographical origin of the provenances and the growing conditions including climate (rainfall and temperature), irrigation water, soil, and fertilizers, etc. (Djerrad et al., 2015; Tohidi et al., 2017; Bistgani et al., 2018). The properties of pepper cultivation such as climate and soil are also important in affecting antioxidants in pepper essential oils and worth future study. The relationships between these antioxidants and habitats might also be further verified by the same species transplantation studies. It would be better to analyze more samples with improved statistical analysis to establish which pepper has better positive effects.

Last but not least, as for our hypothesis “White and black, which one is better?”, there are still some limitations of the present study. Our research has been only carried out on essential oils. However, besides oils, these two peppers are also often used in powder form. Therefore, the water extracts of these peppers should also be compared, whose antioxidant activities should be evaluated in a future study. On the other hand, the anti-inflammatory activities of black and white pepper should also be analyzed in our future work to correlate with the present work. In addition, the DNA damage investigation under *in vitro* study should also be done for future work. All these could help to provide more information and a better understanding of these two peppers.

## Conclusion

This comparative study on the intracellular and *in vitro* antioxidant activities of essential oil from white and black pepper (*Piper nigrum* L.) proves that pepper essential oils exhibit a good antioxidant effect owing to their active components. Black pepper essential oil is always slightly more effective than white pepper essential oil. This is probably explained by the fact that the most abundant components of BPEO and WPEO are 3-carene and caryophyllene, respectively, and the scavenging abilities of the former are more effective than those of the latter. Generally, pepper essential oil is a mixture and always has stronger antioxidant activities and free radical scavenging potentials than single components, indicating that pepper essential oil is a broad-spectrum natural antioxidant with important application prospects. Meanwhile, as a natural antioxidant, its function is sometimes better than synthetic antioxidants, suggesting the potential use of this natural antioxidant in food processing and its benefits for human health.

## Data Availability

The original contributions presented in the study are included in the article/Supplementary Material, further inquiries can be directed to the corresponding author.
