# Supplementary material for: Comparative Analysis of Intracellular and in vitro Antioxidant Activities of Essential Oil From White and Black Pepper (Piper nigrum L.)
Source: Front Pharmacol. 2021 Jun 25;12:680754. doi: 10.3389/fphar.2021.680754 (PMC8267920; doi:10.3389/fphar.2021.680754)
Supplement: Supplementary file 1 [file Table1.DOCX]

**Table 1** EC_50_ values (mg/mL) of WPEO, BPEO, synthetic antioxidants (BHT, PG and Vc) and seven standards (SR: Superoxide radical scavenging activity; HR: Hydroxyl radical scavenging activity; DR: DPPH radical scavenging activity; ILLP: Inhibition of lipoprotein lipid peroxidation; WPEO: White pepper essential oil; BPEO: Black pepper essential oil; BHT: Butylated hydroxytoluene; PG: Propylgallate; Vc: Ascorbic acid)

| Antioxidants | | EC_50_ values (mg/mL) | | | |
| --- | --- | --- | --- | --- | --- |
|  |  | SR | HR (×10^-5^) | DR | ILLP |
| Natural | WPEO | 0.437 | 0.486 | 7.332 | 0.688 |
|  | BPEO | 0.327 | 0.204 | 6.348 | 0.624 |
| Synthetic | BHT | 0.591 | 0.371 | 2.594 | 0.018 |
|  | PG | 1.256 | 1.564 | 0.719 | 0.125 |
|  | Vc | 0.401 | 7.297 | 1.416 | 1.121 |
| Standards | α-pinene | 0.550 | 0.843 | 10.201 | 0.819 |
|  | β-pinene | 0.473 | 1.169 | 10.272 | 1.025 |
|  | 2-carene | 0.457 | 2.173 | 10.274 | 1.094 |
|  | 3-carene | 0.412 | 0.710 | 10.062 | 0.751 |
|  | Limonene | 0.291 | 4.490 | 9.720 | 0.848 |
|  | Linalool | 0.637 | 0.557 | 10.414 | 0.619 |
|  | Caryophyllene | 0.727 | 0.779 | 10.554 | 0.786 |
