# Supplementary figures and images for: Comparative Analysis of Intracellular and in vitro Antioxidant Activities of Essential Oil From White and Black Pepper (Piper nigrum L.)
Source: Front Pharmacol. 2021 Jun 25;12:680754. doi: 10.3389/fphar.2021.680754 (PMC8267920; doi:10.3389/fphar.2021.680754)

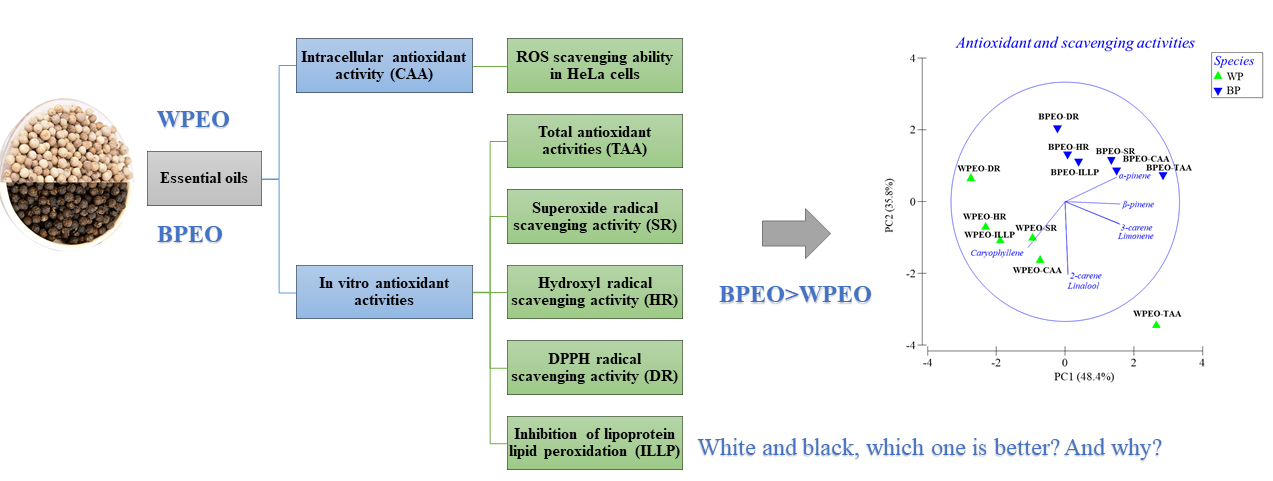

Supplement: Supplementary file 2 [file Image1.PNG]
