# Supplementary material for: Comparative Analysis of Intracellular and in vitro Antioxidant Activities of Essential Oil From White and Black Pepper (Piper nigrum L.)
Source: Front Pharmacol. 2021 Jun 25;12:680754. doi: 10.3389/fphar.2021.680754 (PMC8267920; doi:10.3389/fphar.2021.680754)
Supplement: Supplementary file 3 [file DataSheet1.DOCX]

Supplementary Material

# Supplementary Figures and Tables

## Supplementary Figures

**Supplementary Figure 1.** The scavenging superoxide anion activity (SR) of WPEO, BPEO and synthetic antioxidants (BHT, PG and Vc) (A) and seven standards (B). (Data are reported as the mean ± SD of three replicates. Bars with different letters are significantly different at p ≤ 0.05 according to one-way ANOVA; WPEO: White pepper essential oil; BPEO: Black pepper essential oil; BHT: Butylated hydroxytoluene; PG: Propylgallate; Vc: Ascorbic acid)

**Supplementary Figure 2.** The scavenging hydroxyl radical activity (HR) of WPEO, BPEO and synthetic antioxidants (BHT, PG and Vc) (A) and seven standards (B). (Data are reported as the mean ± SD of three replicates. Bars with different letters are significantly different at p ≤ 0.05 according to one-way ANOVA; WPEO: White pepper essential oil; BPEO: Black pepper essential oil; BHT: Butylated hydroxytoluene; PG: Propylgallate; Vc: Ascorbic acid)

**Supplementary Figure 3.** The scavenging DPPH radical activity (DR) of WPEO, BPEO and synthetic antioxidants (BHT, PG and Vc) (A) and seven standards (B). (Data are reported as the mean ± SD of three replicates. Bars with different letters are significantly different at p ≤ 0.05 according to one-way ANOVA; WPEO: White pepper essential oil; BPEO: Black pepper essential oil; BHT: Butylated hydroxytoluene; PG: Propylgallate; Vc: Ascorbic acid)

**Supplementary Figure 4.** The inhibition of lipoprotein lipid peroxidation activity (ILLP) of WPEO, BPEO and synthetic antioxidants (BHT, PG and Vc) (A) and seven standards (B). (Data are reported as the mean ± SD of three replicates. Bars with different letters are significantly different at p ≤ 0.05 according to one-way ANOVA; WPEO: White pepper essential oil; BPEO: Black pepper essential oil; BHT: Butylated hydroxytoluene; PG: Propylgallate; Vc: Ascorbic acid)

## Supplementary Tables

**Supplementary Table 1.** Information of the seven standards from WPEO and BPEO (Relative content of the standards citied from our previous results (WANG et al., 2018); WPEO: White pepper essential oil; BPEO: Black pepper essential oil; Data are reported as the mean ± SD of three replicates)

| Standards | Molecular formula | Structures | Relative content (%) | |
| --- | --- | --- | --- | --- |
|  |  |  | WPEO | BPEO |
| α-pinene | C_10_H_16_ | 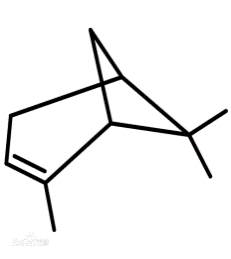 | 5.2 ± 0.2 | 8.6 ± 0.4 |
| β-pinene | C_10_H_16_ | 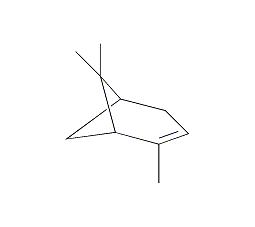 | 9.0 ± 0.3 | 14.0 ± 0.5 |
| 2-carene | C_10_H_16_ | 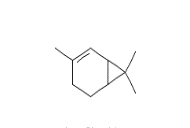 | 1.1 ± 0.4 | 0.1 ± 0.3 |
| 3-carene | C_10_H_16_ | 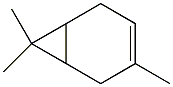 | 25.1 ± 1.1 | 33.2 ± 1.0 |
| Limonene | C_10_H_16_ | 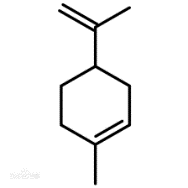 | 16.0 ± 0.4 | 19.2 ± 1.3 |
| Linalool | C_10_H_18_O | 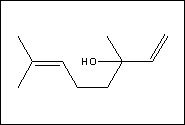 | 0.4 ± 0.2 | 0.1 ± 0.1 |
| Caryophyllene | C_15_H_24_ | 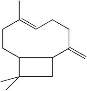 | 33.4 ± 0.5 | 13.0 ± 1.8 |
